# Long-stay in short-stay inpatient facilities: risk factors and barriers to discharge

**DOI:** 10.1186/1471-2458-9-306

**Published:** 2009-08-22

**Authors:** Antonella Gigantesco, Giovanni de Girolamo, Giovanni Santone, Rossella Miglio, Angelo Picardi

**Affiliations:** 1 Mental Health Unit, Center of Epidemiology, Health Surveillance and Promotion, Italian National Institute of Health, Roma, Italy; 2 Psychiatric Unit, United Hospitals of Ancona & Polytechnic University of Marche, Ancona, Italy; 3 Department of Statistics, University of Bologna, Bologna, Italy; 4 Fatebenefratelli Hospitalization and Care Scientific Institute, Brescia, Italy

## Abstract

**Background:**

The aim of the present study was to assess the characteristics of long-stay inpatients in public and private Italian acute inpatient facilities, to identify risk factors and correlates of the long duration of hospital stay in these patients, and to identify possible barriers to alternative placements.

**Methods:**

All patients in 130 Italian public and private psychiatric inpatient units who had been hospitalized for more than 3 months during a specific index period were assessed with standardized assessment instruments and compared to patients discharged during the same index period, but staying in hospital for less than 3 months (short-stay inpatients). Assessed domains included demographic, clinical, and treatment characteristics, as well as process of care. Logistic regression analysis was used to identify specific variables predicting inpatient long-stay status. Reasons for delaying patient discharge, as reported by treatment teams, were also analyzed.

**Results:**

No overall differences between long-stay and short-stay patients emerged in terms of symptom severity or diagnostic status. Admission to a private inpatient facility and display of violent behavior during hospital stay were the most powerful predictors of long-stay. Lack of housing and a shortage of community support were the reasons most commonly cited by treatment teams as barriers to discharge.

**Conclusion:**

Extra-clinical factors are important determinants of prolonged hospitalization in acute inpatient settings.

## Background

In most countries' health care systems, the use of acute hospital beds represents an important economic and policy issue: hospital care commonly takes up a considerable share of mental health budgets, and cost reduction efforts thus far have focused on shortening hospital stays to the briefest amount of time possible. Some authors, however, have linked potentially higher readmission risks to shorter length of stay [1,2]. Hence, a large body of empirical research has attempted to estimate the current extent of inappropriate bed use. Data collection systems have been developed to describe trends in hospitalization rates, re-hospitalization levels, and average length of stay, as well as to identify factors affecting hospital stay duration. Findings from these studies, however, have been inconsistent: for example, although several studies report longer length of stay for older, female patients, other studies have shown that demographic variables are poor length-of-stay indicators [3].

The role of psychiatric diagnosis is also unclear: for instance, some studies suggest that diagnosis is not associated with length of stay, but others have found that psychoses or major depression [4] are correlated with longer length of stay. Moreover, clinician based measures, such as positive symptoms subscale scores on the Brief Psychiatric Rating Scale (BPRS), have shown evidence of being valid length-of-stay predictors [3].

In Italy all mental hospitals have gradually shut-down, and mental health care is now delivered through a network of community-based mental health services. In particular, acute inpatient care is provided by a network of public and private facilities [5].

Inpatient public facilities are represented by 262 General Hospital Psychiatric Units (GHPUs), 23 University Psychiatric Clinics (UPCs), 16 Community Mental Health Centers operating 24 hours a day (24-hr CMHCs), and 14 crisis centers or medical wards with few beds available for patients with acute mental disorders. Overall, public facilities in Italy have a total of 4,108 beds, with 0.78 beds per 10,000 inhabitants. Fifty-four private inpatient facilities (with a total of 4,862 inpatient beds, mean size: 90 ± 48.2 beds) are also in operation, with 0.94 beds per 10,000 inhabitants. The Italian National Health Service fully covers the cost of stay in all public and private facilities. In most regions, patients are free to choose between the public and private systems for inpatient admission, although admission generally occurs through psychiatric referrals made in CMHCs which deliver the full spectrum of care for geographically defined catchment areas. The Italian psychiatric inpatient care system can therefore be defined as a mixed, competitive system. As in other countries, various factors – such as more effective forms of intervention, the development of community-based residential facilities for severe patients requiring long-term care, and policy-makers' focus on cost containment – have progressively led to briefer lengths of stay in acute Italian inpatient facilities. Yet, a recent national survey of all public and private inpatient facilities [5] found considerable variation in average length of stay for patients admitted to different public and private inpatient facilities. The highest average length of stay was observed in private facilities (39.7+17.8 days), followed by 24-hr CHMCs (37.0+55.3 days) and UPCs (18.5+7.1 days); GHPUs showed the shortest average length of stay (12.0+3.4 days).

Differences in length of stay are due to the roles played by different facilities in the overall Italian mental health care system, while no specific guidelines concerning length of stay have been issued by national or local health authorities. For example, shorter length of stay in GHPUs is due to a variety of factors, including the low number of acute public inpatient beds per 10,000 inhabitants [5], with the need of a rapid turn-over of inpatients, the different casemix of patients admitted to publics versus private facilities and the availability of local private inpatient facilities, where patients requiring longer stay could be transferred.

Median values also showed considerable across facility differences; moreover, substantial proportions of inpatients with much longer hospital stays were observed; for instance, in the Lazio Region (one of Italy's most densely populated regions), 10% of patients admitted to private facilities in the year 2005 had stayed there more than four months [6]. These data suggest that a sizeable group of patients continues to have prolonged hospital stays. It is therefore important to clarify the degree to which this phenomenon corresponds to patients' actual needs, or whether it depends on care system shortcomings or on difficulties in managing patients presenting the most difficult and complex clinical situations.

The present study was conducted in the framework of the PROGRES Acute project – a 2-phase national survey jointly launched by the Italian National Institute of Health and the Department of Mental Health of Trieste to obtain data on the physical characteristics, activity data, demographic and clinical characteristics of residents, staffing arrangements, regional provision of care, discharge rates, and admission rules of all Italian public and private acute inpatient facilities.

In the second phase of the PROGRES Acute project a random sample of facilities was selected, and a representative sample of inpatients staying in the selected facilities was individually evaluated; this exercise represented an excellent opportunity to shed light on the subgroup of inpatients hospitalized at length. The present paper aimed at assessing the sociodemographic, clinical, and treatment-related characteristics of long-stay inpatients in Italian public and private acute facilities. The study also attempted to clarify the barriers preventing or delaying patient discharge from acute inpatient facilities.

## Methods

### Sampling

All 21 Italian regions, with the exception of Sicily, participated in the study. Each region designated a coordinator to organize and supervise data collection. Phase 1 surveyed the physical characteristics, staffing arrangements, admission rules, and activities of all public and private acute inpatient facilities [5]. In phase 2, a 20% random sample of GHPUs (stratified by Region) and all remaining public and private facilities surveyed in phase 1 were initially selected for a more in-depth study. Severe financial constraints at the time in the Lazio Region limited its participation to a survey of facilities available to provide data on a voluntary basis. At the same time, however, eight, not originally sampled GHPUs asked to participate and were included in the study.

Furthermore, organizational problems prevented the participation of six 24-h CMHCs, three UPCs, and 18 private facilities. Phase 2 therefore examined 130 public facilities and 36 private facilities.

The study sample comprised all inpatients with a length of stay of more than 90 days and no planned discharge ("long-stay inpatients"), and all inpatients staying for 90 days or less with a planned discharge ("short-stay inpatients"), in public and private facilities surveyed during an index period of 12 and 3 days, respectively. The shorter, 3-day index period was used for private facilities, because the National Association of Private Hospitals consented to patient recruitment and evaluation for only a limited number of days, due to time and work constraints in these facilities. Given that the study focused on factors associated with long- vs. short-term inpatient stays, patients admitted and discharged during these time periods were not considered for inclusion. The final sample therefore included 127 long-stay and 870 discharged (or planned discharge) inpatients.

### Assessment

All regional coordinators were trained in the administration of the study instruments. In larger regions, additional research assistants participated in data collection under the coordinators' close supervision. A researcher visited each facility and completed standardized assessment for each patient included in the study. Socio-demographic information was retrieved from patient records. An ICD-10 primary diagnosis was assigned by the patients' treating physician. Detailed information on patients' behavior and psychosocial interventions received was also obtained. Assessment was based on the 24-item BPRS [7] and on the Personal and Social Performance scale (PSP). The PSP is a modified validated version of the Social and Occupational Functioning Assessment Scale (SOFAS) [8]. As with the SOFAS scale, PSP scale scores range from 100 (excellent functioning) to 1 (extremely severe impairment with risk for survival). With reference to long-stay inpatients only, treatment teams were asked to express their opinions on the reasons for delayed discharge; the responses were then coded into the following non mutually exclusive categories: presence of serious physical illness, lack of independent or staffed accommodation (placement not available after discharge approval by hospital staff), lack of economic resources, lack of close relationships and social support, risk of antisocial behavior, and lack of treatment response.

### Data and statistical analysis

The first analysis used descriptive statistics (Student's t-test or Chi-square test, as appropriate) to examine the characteristics of long-stay vs. short-stay inpatients and to summarize the information about reasons contributing to the prolonged stay.

In the second stage, a multiple logistic regression model was developed to investigate relations among inpatients' socio-demographic and clinical characteristics, violent behaviour, psychosocial interventions received occurring during hospitalization, and long-stay status. The following criteria were used to classify psychosocial interventions: (1) 'psychotherapy' was considered any individual supportive, dynamic, or cognitive-behavioral psychotherapy; or any group dynamic, cognitive-behavioral, or psycho-educational therapy/intervention; (2) 'rehabilitative treatment' was any form of individual or group social skills training; and (3) 'family education treatment' referred to any type of information and counseling intervention aimed at providing patients and their family members with information on mental disorders and available treatments. 'Violent behavior' was considered any incident occurring during hospitalization, in which a patient had attempted to physically harm others (e.g., hospital staff members, other patients, or visitors) or to damage property.

A set of sociodemographic, clinical, and treatment-related variables were entered into the regression model as independent variables, and long-stay in-patient status (no vs. yes) was entered as the dependent variable. The Odds Ratio (OR) with 95% Confidence Interval (CI) was used as a measure of association.

Given that dichotomous variables yield limited information on psychosocial interventions, the proportion of inpatients receiving more or less than 5 sessions of any type of psychosocial intervention was also calculated and examined in further analyses.

### Ethical issues

Ethical approval is granted for this study from the Italian Ministry of Health which approved and financed the PROGRES-Acute Project.

## Results

Table 1 shows the main sociodemographic and clinical characteristics of long-stay inpatients. Overall, the long-stay sample consisted of middle-aged patients, living either with a partner or with family members and who were generally unemployed or receiving a social- or disability pension.

**Table 1:** Sociodemographic and Clinical Characteristics of Acute Inpatients

		**Long-stay inpatients (N = 127)**		**Short-stay inpatients** **(N = 870)**		**Statistics**	
		**Mean (SD)**		**Mean (SD)**		t	**P**
**AGE**	Mean	49.9 (15.2)		45.6 (15.2)		3.0	0.003
		**N**	**%**	**N**	**%**	**x**^2^	**p**
**GENDER**	Male	67	53.0	439	50.5	0.23	0.64
	Female	60	47.0	431	49.5		
**MARITAL STATUS**	Single	82	65.0	426	49.0	15.8	< 0.001
	Married	17	13.0	257	30.0		
	Separated or divorced or widowed	28	22.0	179	21.0		
**EDUCATION**	≤ 8 years	73	57.0	588	72.0	9.1	0.003
	> 8 years	52	41.0	232	28.0		
**OCCUPATIONAL STATUS**	Currently unemployed	33	26.0	217	26.0	20.3	< 0.001
	Full/part-time/temporary work/vocational training	19	15.0	203	23.0		
	Social or disability pension	63	50.0	265	34.0		
	Other (e.g., housewife, student, etc)	11	9.0	150	17.0		
**NATIONALITY**	Italian	126	99.0	848	98.0	1.85	0.60
	European Union or Other	1	1.0	12	2.0		
**LIVING SITUATION**	At home						
	- Alone	22	17.0	160	19.0	16.1	0.001
	- With partner or relatives or friends	77	61.0	609	72.0		
	Institution	16	13.0	54	6.0		
	Other	11	9.0	28	3.0		

Table 2 shows the sample's clinical characteristics. The majority had received a diagnosis of schizophrenic or mood disorder. Long-stay patients had been in acute facilities for a median of 175 days (range: 91-2,873; 75th percentile: 355). Significant differences between the two inpatient groups were observed in positive, negative, and disorganization scores, as measured by the relevant BPRS items and by the PSP total score (Table 2).

**Table 2:** Clinical Characteristics of the Sample

	**Long-stay inpatients (N = 127)**		**Short-stay inpatients** **(N = 870)**		**Statistics**	
	**N**	**%**	**N**	**%**	**x**^2^	**p**
**Diagnosis**					24.6	< 0.001
*Anxiety disorder*	1	1.0	38	4.0		
*Organic psychiatric disorder*						
*including mental retardation*	14	12.0	46	5.0		
*Schizophrenia*	59	51.0	305	35.0		
*Mood disorder*	26	22.0	316	37.0		
*Personality and substance use disorder*	16	14.0	160	19.0		
**Admission Status**					9.5	0.009
*Voluntary*	121	95.3	759	87.2		
*Compulsory*	4	3.1	104	12.0		
*Unknown*	2	1.6	7	0.8		
**Patient's attitude at admission**					44.3	< 0.001
*Hostile*	14	11.0	146	16.8		
*Unwilling*	17	13.4	140	16.1		
*Indifferent*	26	20.5	133	15.3		
*Favorable*	60	47.2	446	51.3		
*Unknown*	10	7.9	5	0.6		
**Insight**						< 0.001
*No insight*	40	31.5	153	17.6	56.6	
*Partial insight with poor compliance*	24	18.9	214	24.6		
*Good insight and compliance*	53	41.7	498	57.2		
*Unknown*	10	7.9	5	0.6		
**Total**						
**BPRS score**	**N**	**Mean** **(SD)**	**N**	**Mean** **(SD)**	**t**	**P**
**BPRS total**	120	53.1 (20.8)	756	45.9 (15.3)	3.6	< 0.001
**BPRS positive symptoms**	124	7.9 (5.4)	813	6.0 (3.8)	3.7	< 0.001
**BPRS negative symptoms**	124	10.5 (5.7)	813	8.2 (4.4)	4.3	< 0.001
**BPRS disorganization symptoms**	124	6.2 (4.3)	813	4.8 (2.8)	3.5	0.001
**BPRS anxiety-depression symptoms**	124	10.8 (4.8)	813	11.3 (4.9)	1.0	0.324
**BPRS manic symptoms**	124	53.1 (20.8)	813	6.0 (3.6)	0.74	0.463
**PSP total score**	119	41.8 (19.1)	781	52.5 ± 19.1	5.7	< 0.001

### Reasons contributing to prolonged hospital stay and treatments received

Table 3 summarizes the reasons given by treatment teams to most likely factors contributing to the patients' prolonged hospital stays. They reflected the opinions of the staff who better knew clinical and socioeconomic status of patients. Lack of housing and community support were the most frequently cited reasons preventing discharge. The presence of a concomitant serious physical disorder was very uncommon. Surprisingly, few patients reported financial difficulties (with the exception of lack of housing). Approximately one third had no close relationships or social support, and approximately four out of ten were at rather high risk of antisocial behavior at discharge.

**Table 3:** Reasons Contributing to Prolonged Stays, as Reported by Patient Treatment Teams *(Categories not Mutually Exclusive)*

	**N of long-stay inpatients**	**% of the total number of long-stay inpatients**
**Lack of independent or staffed accommodation**	72	57.0
**Lack of social support**	46	36.0
**Risk of antisocial behaviors at discharge**	43	34.0
**Lack of response to treatment**	38	30.0
**Lack of economic resources**	13	10.0
**Presence of physical illness**	11	9.0

As to treatment received by long-stay inpatients, rehabilitative interventions were provided for 24%, and family education for 35% of these in-patients. Of the 30 long-stay inpatients who had received rehabilitative intervention, 23 had received more than 5 sessions; and 24 of the 45 inpatients and their families who had received family psychoeducation had participated in more than 5 sessions (table 4).

**Table 4:** Number of Psychological Therapy Sessions and Rehabilitative Interventions Delivered in Acute Inpatient Facilities

	**Long-stay inpatients**		**Short-stay inpatients**	
	1-5 N (%)	More than 5 N (%)	1-5 N (%)	More than 5 N (%)
Individual supportive psychotherapy	12 (9.0)	41 (32.0)	224 (26.0)	137 (16.0)
Individual dynamic psychotherapy	2 (2.0)	0 (0.0)	23 (3.0)	7 (1.0)
Individual cognitive-behavioral therapy	1 (1.0)	6 (5.0)	18 (2.0)	10 (1.0)
Group dynamic psychotherapy	1 (1.0)	3 (3.0)	38 (4.0)	23 (3.0)
Group cognitive-behavioral therapy	1 (1.0)	12 (9.0)	25 (3.0)	15 (2.0)
Group social skills training	7 (5.0)	23 (18.0)	50 (6.0)	29 (3.0)
Group psychoeducation	6 (5.0)	2 (2.0)	28 (3.0)	12 (1.0)
Family education	21 (16.0)	24 (19.0)	236 (27.0)	54 (6.0)

### Multivariate analysis

As shown in Table 5, a logistic regression analysis identified the following correlates of long-stay status: admission to a private facility, violent behavior, poor personal and social functioning, unmarried status, older age, higher education and receipt of rehabilitative intervention. No association emerged between long-stay status and symptom severity. The strongest long-stay predictor by far was private in-patient status, which presented an odds ratio as high as approximately 10, followed by violent behavior, with an odds ratio of 4.5.

**Table 5:** Variables Associated With Long-Stay Inpatient Status in Public and Private Italian Acute Psychiatric Units (N = 997)

**Variables**		*P*-value (Wald test)**	**Adjusted Odds Ratio (95%CI)**
**Age**		*P *< 0.05	1.02 (1.00-1.04)
**Gender**	Female	NS	0.99 (0.59-1.68)
	Male*		
**Marital status**	Single/divorced/widowed Married*	*P *< 0.05	2.43 (1.20-4.94)
**Education**	> 8 years	*P *< 0.05	2.03 (1.18-3.50)
	≤ 8 years*		
**Diagnosis**	Organic disorder	NS	5.66 (0.58-55.18)
	Schizophrenic psychosis disorder	NS	6.89 (0.79-60.23)
	Mood disorder	NS	2.63 (0.31-22.48)
	Personality or substance abuse disorder	NS	2.86 (0.32-25.70)
	Anxiety disorder*		
**BPRS score**			
	Positive symptoms	NS	0.99 (0.92-1.07)
	Negative symptoms	NS	1.05 (0.98-1.13)
	Disorganization	NS	0.94 (0.85-1.05)
	Anxiety, Depression symptoms	NS	1.00 (0.95-1.06)
	Manic symptoms	NS	1.02 (0.94-1.11)
**PSP score**		*P *< 0.01	0.97 (0.96-0.99)
**Violent episodes**	Yes	*P *< 0.01	4.51 (1.74-11.70)
	No*		
**Psychotherapeutic treatment**	Yes	NS	1.02 (0.58-1.79)
	No *		
**Rehabilitative treatment**	Yes	*P *< 0.01	2.58 (1.36-4.91)
	No *		
**Family education treatment**	Yes	NS	1.44 (0.81-2.58)
	No *		
**Type of service**	Private	*P *< 0.001	9.98 (5.59-17.81)
	Public*		

*Reference categories

**NS indicates not statistically significant

## Discussion

The PROGRES-Acute survey is the first study ever carried in Italy aimed at obtaining comprehensive nationwide data on public and private inpatient facilities and their functioning. It provides valuable information about the phenomenon of long-stay inpatients hospitalized in acute facilities, which represents an important problem for the overall effectiveness of mental health services (also from a financial perspective). The main findings and most important clinical and treatment implications of this study are discussed here below.

### Variables associated with hospital long-stay

Several studies have previously reported length-of-stay associations with patient age [9] and gender characteristics [10]. Our results are consistent with these findings in terms of age, but not gender. It is possible that gender *per se *is not a key long-stay risk factor and that other confounding factors, such as the specific clinical characteristics of study participants and situational variables, have a greater impact on hospital stay duration [11].

Our study also did not replicate previous findings showing an association between long-stay status and diagnosis: based on ICD-10 diagnostic categories, and controlling for other variables, no significant differences between long-stay and short-stay inpatients emerged. We also observed no association with patients' severity in terms of psychopathology. Although severity of psychopathology has been found to influence length of stay [3], the issue of how best to develop valid and reliable ways to measure illness severity has always represented a challenge for the field [12]. Various serious proposals in this regard have emerged in recent years; our study used the 24-item BPRS, an expanded version of the 18-item BPRS, with defined scale points and probe questions, which has shown improved inter-rater reliability over previous versions.

An high percentage of short-stay patients was found among compulsorily admitted subjects. This is consistent with data found by other authors [11], and is probably due to the fact that a "short" duration of admission (up to 90 days in our study) was sufficient for controlling acute psychiatric symptoms, while long-stay status is determined by variables other than symptomatology (as shown also by the results of the multivariate analysis).

The other variables examined herein have also been investigated by other authors, and our finding that unmarried status is associated with long hospital stay is consistent with results from several previous studies [3,4,11].

Although we found that poor psychosocial functioning was positively associated with long-stay status, it is generally unclear whether more impaired patients have longer lengths of stay due to difficulties in planning discharges and in community resettlement efforts, or whether hospital stay leads to a loss of basic daily living skills and impairment in ability to function in social environments [13]. Of course, it is possible that both factors influence long-stay: patients with poor psychosocial functioning tend to remain in hospital longer, and this situation in turn facilitates the further loss of daily living skills, increases dependence, and weakens social networks.

An intriguing finding was that patients with higher education level (more than 8 years) were hospitalized for longer than those with lower education level. However, an high school degree (or beyond) was also found in the majority (60%) of long-stay patients in a North-American study [14], while in a German sample of inpatients subjects who had failed to attain a grammar school certificate were more readily discharged as compared to inpatients with an high school degree [11]. An hypothetic explanation may be that treatment psychiatrists had higher expectations of improvement about patients with higher education, and a longer stay of those patients was related to the tendency of these physicians to not discharge patients who still did not show a clinically significant improvement.

### Length of stay and hospital setting

The strongest predictor of long hospital stay was admission to a private inpatient facility. It should be noted that this is the first national survey ever conducted in Italy to include patients admitted to private inpatient facilities. Indeed, very few investigations at an international level have examined inpatients admitted to private facilities. It can be argued that the tendency to longer stay in private inpatient facilities is due to specific differences in their functioning and staffing arrangements. In fact, private Italian hospital facilities, vs. public facilities, present several characteristics found to be associated with longer length of stay [5], such as lower staff-patient ratio, poorer coordination with community mental health services (although referral to community services may be associated with higher rehospitalization risk, or does not necessarily reduce the risk of rehospitalization [2,15]), and a higher number of beds [16].

### Risk of violent behavior and long-stay

Our finding that long-stay inpatients were more likely to display violent behaviors during hospitalization has also been reported previously [17]. Various explanations can account for this association: duration of hospital stay may be determined by the violence a patient exhibits. Alternatively, long-term hospitalization itself may contribute to the manifestation of violent behavior – perhaps due to patient frustration about a delayed discharge. Studies conducted in other countries have shown that violent behavior during hospitalization is most likely linked to factors other than long-term hospitalization.

Specifically, acute symptom severity and prior history of dangerousness appear to be the strongest predictors for disruptive behavior in psychiatric units [18,19]. Patients in the present study frequently had their stay prolonged because they were judged to be at risk of antisocial behavior. Professionals tend to be cautious when placing patients who have exhibited violent behavior in less-supervised settings, in an attempt to avoid triggering events with undesirable consequences. Perhaps the team members in our study tended to carefully weight the risks of discharging patients into family environments, and these patients therefore remain hospitalized for longer periods. This finding has direct implications for the complex ward management of patients showing violent behavior, because they should receive more intensive management during their hospital stay. Yet, as observed elsewhere [20], Italian psychiatric units rarely provide intensive case management and direct behavioural forms of intervention – such as social skills training -which is specific to the post-discharge environment and helps patients better cope with stress and anger. This general scenario persists, in spite of wide-scale acknowledgment of the importance of the inclusion of these programmes in individual inpatient care plans [21].

### Treatments and long-stay

The findings about process of care were more complex and difficult to interpret. On one hand, psychotherapies that can require prolonged treatments did not predict prolonged hospital stay. On the other, long-stay inpatients (vs. discharged patients), as expected, were more likely to have received rehabilitative intervention that can require longer hospitalization. In any event, it is worth noting that rehabilitative interventions were delivered to only 24% of these inpatients – a finding that is reason for concern, because 50% of them stayed in hospital for approximately 5 months, and 25% stayed for more than 11 months. Moreover, these findings raise the question of why these patients were not moved from hospital- to community-based settings, including residential facilities.

Staff members noted that many patients continued to occupy acute hospital beds due to the unavailability of appropriate alternative placements – a problem found to be linked to inappropriate bed use in acute settings, in other studies conducted in European countries with similar financing and health care delivery systems. The issue is pressing in the UK, where high proportions of 'new long-stay patients' tend to receive inappropriate, continued acute care, following the country's historical shift to community care [22]. Some of these patients merely require home-based community support, such as group homes. The situation is similar in Italy, where poorly suitable post-discharge accommodation inevitably places greater pressure on acute psychiatric beds. For example, just one out of six residential facilities occasionally admits patients with acute illness episodes [2].

Residential rehabilitative facilities should therefore be made available to patients requiring more intense levels of care. Yet, in Italy, beds in these facilities are not easily made vacant, as shown in a recent nationwide study on Italian psychiatric residential facilities [23], which found that resident turnover was extremely low. These findings point to the need for the development of sufficient and appropriate alternative care settings for patients admitted to acute psychiatric units.

## Conclusion

The present study has a number of limitations. Firstly, information on treatment and intervention methods was provided by treatment staff, and it was not possible to check the accuracy of these responses. We were also unable to assess the appropriateness of the reported interventions.

We may think that it is more probable to recruit short-stay inpatients in public facilities, and therefore the percentage of long-stay inpatients is smaller in this kind of facilities, but the different duration of the recruitment period does not explain the remarkable different length of stay found in the two types of facilities. In another paper [5] we have shown that the average length of stay in public General Hospital Psychiatric Wards was 12.0 (+3.4) days as compared to 39.7 (+17.8) days in private facilities with median value 11.4, 37.6 respectively.

Another limitation lies in the cross-sectional design of the survey, which does not allow for the drawing of causal inferences on the determinants of this complex phenomenon. Difficulties in generalizing the results of this survey to other countries may be related to differences in study populations and settings. Nevertheless, with few exceptions, the study findings are generally consistent with the findings of previous studies conducted worldwide.

In conclusion, our findings suggest that for many patients it is not mental disorder alone, but a combination of behavioral and functioning factors that leads to prolonged hospitalization. For example, patients with marked functional impairment likely find placement in acute hospital units, because this setting can provide the assistance they require in daily living activities. Clearly, other types of residential facilities could provide this type of service, but the co-occurrence of undesirable behaviors in some individuals can make this type of placement difficult for all involved.

Hence, the inadequacy or lack of community placements or management factors for long-stay inpatients shows evidence of leading to unnecessarily longer and more costly hospital stays, reducing resources available for critically ill patients thereby. In summary, acute care hospitals may have longer-term admissions due to clinical indications such as manifestations of dangerous behavior, to system factors such as lack of services organization, or to the unavailability of specialized programs and approaches for this population. It might be useful to increase the availability of care in community-based residential facilities (though the cost effectiveness of this approach should be demonstrated). It is also important to implement more activities targeted at helping patients integrate into their local communities, such as supported housing programs and/or family involvement in therapy. For patients with needs requiring longer inpatient care due to dangerous behavior, appropriate policies, such as creating anger management programs and specialized units, are recommended.

## Competing interests

The authors declare that they have no competing interests.

## Authors' contributions

AG participated in the design of the study, conceived of and performed the analyses and drafted the manuscript, GdG participated in the design and coordination of the study and helped to draft the manuscript, GS helped to draft the manuscript, RM quality-checked the statistical analyses, AP specified study design and helped to draft the manuscript. All authors read and approved the final manuscript.

## Pre-publication history

The pre-publication history for this paper can be accessed here:


